# Cost‐effectiveness of closed incision negative pressure wound therapy in preventing surgical site infection among obese women giving birth by caesarean section: An economic evaluation (DRESSING trial)

**DOI:** 10.1111/ajo.13677

**Published:** 2023-05-18

**Authors:** Jennifer A. Whitty, Adam P. Wagner, Evelyn Kang, David Ellwood, Wendy Chaboyer, Sailesh Kumar, Vicki L. Clifton, Lukman Thalib, Brigid M. Gillespie

**Affiliations:** ^1^ Norwich Medical School University of East Anglia Norwich UK; ^2^ National Institute for Health Research (NIHR) Applied Research Collaboration (ARC) East of England (EoE) Cambridge UK; ^3^ National Health and Medical Research Council Centre of Research Excellence in Wiser Wound Care, Menzies Health Institute Griffith University Gold Coast Queensland Australia; ^4^ Gold Coast University Hospital, Gold Coast Health Southport Queensland Australia; ^5^ School of Medicine and Dentistry Griffith University Gold Coast Queensland Australia; ^6^ Mater Mothers’ Hospital University of Queensland Brisbane Queensland Australia; ^7^ Mater Research Institute University of Queensland Brisbane Queensland Australia; ^8^ Department of Biostatistics, Faculty of Medicine Istanbul Aydın University Istanbul Turkey

**Keywords:** caesarean section, cost‐effectiveness, economic evaluation, negative pressure wound therapy, surgical site infection

## Abstract

**Background:**

There is growing evidence regarding the potential of closed incision negative pressure wound therapy (ci‐NPWT) to prevent surgical site infections (SSIs) in healing wounds by primary closure following a caesarean section (CS).

**Aim:**

To assess the cost‐effectiveness of ci‐NPWT compared to standard dressings for prevention of SSI in obese women giving birth by CS.

**Materials and Methods:**

Cost‐effectiveness and cost‐utility analyses from a health service perspective were undertaken alongside a multicentre pragmatic randomised controlled trial, which recruited women with a pre‐pregnancy body mass index ≥30 kg/m^2^ giving birth by elective/semi‐urgent CS who received ci‐NPWT (*n* = 1017) or standard dressings (*n* = 1018). Resource use and health‐related quality of life (SF‐12v2) collected during admission and for four weeks post‐discharge were used to derive costs and quality‐adjusted life years (QALYs).

**Results:**

ci‐NPWT was associated with AUD$162 (95%CI −$170 to $494) higher cost per person and an additional $12 849 (95%CI −$62 138 to $133 378) per SSI avoided. There was no detectable difference in QALYs between groups; however, there are high levels of uncertainty around both cost and QALY estimates. There is a 20% likelihood that ci‐NPWT would be considered cost‐effective at a willingness‐to‐pay threshold of $50 000 per QALY. Per protocol and complete case analyses gave similar results, suggesting that findings are robust to protocol deviators and adjustments for missing data.

**Conclusions:**

ci‐NPWT for the prevention of SSI in obese women undergoing CS is unlikely to be cost‐effective in terms of health service resources and is currently unjustified for routine use for this purpose.

## Introduction

Traditionally, surgeons have closed surgical incisions using sutures, staples, tissue glue, paper tape, or a combination, and covered incisions using adhesive film dressings.^1^ There is an increasing number of surgeons, across specialties using closed incision negative pressure wound therapy (ci‐NPWT) following skin closure. Closed incision NPWT is thought to maximise local blood flow,^2^ promote tissue granulation, and reduce risk of haematoma/seroma^3^ or wound dehiscence.^4^ In non‐obstetric operations, ci‐NPWT is increasingly used to reduce wound complication risks, including surgical site infection (SSI), haematoma and seroma, and re‐operation.^5^, ^6^

There is growing evidence regarding ci‐NPWT preventing SSI in healing wounds by primary closure following caesarean sections (CSs).^7^ However, two large trials are equivocal regarding use of ci‐NPWT to prevent SSI in obese women following CS. Tuuli et al. (*n* = 1608) reported no difference in SSI rates (NPWT 3.6% vs standard dressing 3.4%; *P* = 0.70), but a 6.95% (*P* < 0.001) increase in adverse skin reactions in the ci‐NPWT group.^8^ Our recent ADding negative pRESSure to improve healING (DRESSING; *n* = 2035), reported a non‐statistically significant 24% reduction in SSI relative risk (RR), but a higher blistering rate for ci‐NPWT.^9^

Consideration of economic, beyond solely clinical, evidence is important to inform decision‐making. Understanding ci‐NPWT's potential value for money is particularly important given the inconclusive clinical evidence. We conducted a pre‐specified economic evaluation alongside DRESSING to assess cost‐effectiveness of ci‐NPWT compared to standard dressings for SSI prevention in obese women recieving CS.

## Materials and Methods

### Study design

Methods and clinical findings of DRESSING have been reported.^9^, ^10^ DRESSING was a pragmatic, randomised controlled, parallel‐group, superiority trial undertaken in four Australian maternity hospitals. Women with a pre‐pregnancy body mass index of ≥30 kg/m^2^ giving birth by elective or semi‐urgent CS, were stratified by hospital and randomised to receive ci‐NPWT (*n* = 1017) or standard dressing (*n* = 1018). The primary outcome was cumulative incidence of SSI during a 30‐day post‐discharge follow‐up.

This within‐trial economic evaluation was undertaken from the health service perspective. Data on health service resource use and health‐related quality of life (HRQoL; using the SF‐12v2) were collected for the trial duration, which included four‐week follow‐up post‐discharge. No discounting was applied. Method reporting conforms to the Consolidated Health Economic Evaluation Reporting Standards (Table S4).^11^

### Cost and resource use data

Health service resource use during index hospital admission was collected from electronic health records, direct observation, and participant self‐reporting. Resource use related to surgical site management post‐discharge was collected by research nurses through weekly telephone interviews for four weeks post‐discharge. Direct costs (AUD$, 2020) were allocated to each resource using standard costing sources (Table S1).

### Outcome measurement and valuation

Outcomes were evaluated both in terms of SSI avoided (consistent with the trial's primary outcome) and quality‐adjusted life years (QALYs) gained. The SF‐12v2 was measured at trial enrolment, and 1–4 weeks post‐discharge. Utility weights were assigned using the SF‐6D algorithm.^12^, ^13^ QALYs were derived using the area‐under‐the‐curve method with adjustment for baseline utility in subsequent regressions.^14^ For QALY calculation, we used the mean time (11 days) between baseline and first post‐discharge questionnaire, and assumed seven days between subsequent questionnaires.

### Analysis

Data analysis used R. ^15^ Unadjusted comparisons between groups were made for costs and outcomes using available data. Variables were compared between groups using mean and standard deviation (SD), along with mean difference, corresponding 95%CI and *P*‐values.

An intention‐to‐treat (ITT) analysis was used in the base case analysis.^16^ Within R, mice ^17^ was used to explore missingness patterns and conduct multiple imputation (MI; see Appendix S1).^18^ In line with primary analysis from the clinical paper, we singly imputed SSI missingness (primary outcome; missingness: ci‐NPWT = 9; standard = 19) and assumed no SSI for women missing the primary outcome.

#### Cost‐effectiveness and cost‐utility analyses

Cost‐effectiveness was considered through two main analyses. For the cost‐effectiveness analysis (CEA), we consider the difference in total costs between arms compared to the number of SSIs avoided. For the cost‐utility analysis (CUA), cost and QALY data were analysed simultaneously using seemingly unrelated regression (SUR),^19^ allowing for cost and QALY correlation. Both regressions included covariates for arm and site, and the QALY regression additionally included baseline utility.^14^ Resulting arm coefficients estimated the mean difference in cost and effect (QALYs) between groups.

For both CEA and CUA, if one alternative was found both less costly and more effective, it was preferred. Otherwise, an incremental cost‐effectiveness ratio (ICER)^20^ was calculated, comparing ci‐NPWT to standard dressings: within CEA, this corresponds to the incremental cost per SSI avoided; with CUA, this is the incremental cost per QALY. Australia has no explicit cost‐effectiveness threshold; however, interventions with ICERs below $50 000 per QALY are often considered cost‐effective.^21^

Non‐parametric bootstrap resampling^22^ stratified by arm and site was used to give point‐estimates and distributions around differences in costs and outcomes. Bootstrapping used 200 replications from each of 50 imputed data‐sets, giving 10 000 replications.^18^, ^23^ The above specified SURs were fitted to each replicate data‐set. Cost‐effectiveness uncertainty was explored using bootstrap samples to plot: (i) cost‐effectiveness planes, which show estimates of incremental differences in cost and outcome;^22^ and (ii) cost‐effectiveness acceptability curves (CEAC), which show the probability of ci‐NPWT being cost‐effective compared to standard dressings at various ‘willingness‐to‐pay’ thresholds.^24^

#### Sensitivity analyses

Two sensitivity analyses were conducted; for each, bootstrap samples were produced from the 50 imputed data‐sets using the approach described above, having excluded participants as follows:

1. per protocol analysis: subsetting imputed data‐sets to participants defined as ‘per protocol’ in the main paper
2. complete case analysis to check sensitivity to assumptions made regarding missing data: subsetting to participants with complete data.

### Ethics

The study was approved by the ethics committees of the Royal Brisbane and Women's Hospital and Griffith University (HREC/15/QRBW/126; GU: NRS/28/15/HREC). Participants gave informed consent prior to participating in the study.

## Results

### Analysis numbers and missing data

In the base case (ITT) analysis there were *n* = 1017 and *n* = 1018 participants in the ci‐NPWT and standard dressing arms, respectively. The per protocol analysis had *n* = 996 and *n* = 983, while the complete case analysis had *n* = 619 and *n* = 566. Missing data primarily arose from weekly telephone calls post‐discharge (see Appendix S1). Proportion of missing data was significantly different between arms (Fisher's exact test, *P* = 0.0172): ci‐NPWT‐39% vs standard dressing‐44%.

### Health service costs

Table S2 reports costs overall and compared by trial arm. Only intervention costs differed significantly between arms. There are similar costs for standard dressing use in both arms post‐discharge (means: $5.15 vs $5.33; *P* = 0.7178). Index admission is the main cost driver.

For 1251 participants with complete cost data, ci‐NPWT is estimated to cost the health service $162 (95%CI −$170 to $494) per person more than standard dressings (NPWT, mean $13 622, SD $3127; standard dressing, mean $13 460, SD $2855).

### Outcomes

DRESSING reported 75 SSIs in the ci‐NPWT arm and 99 SSIs in standard dressing (RR 0.76, 95%CI 0.57–1.01, *P* = 0.06).^9^ Table S3 reports overall and a comparison by arm of utility values at each time‐point and corresponding QALY scores using the SF‐6D (SF‐12v2). There was no detectable difference between groups on these measures.

### Cost‐effectiveness analysis (CEA)

**Figure 1:**
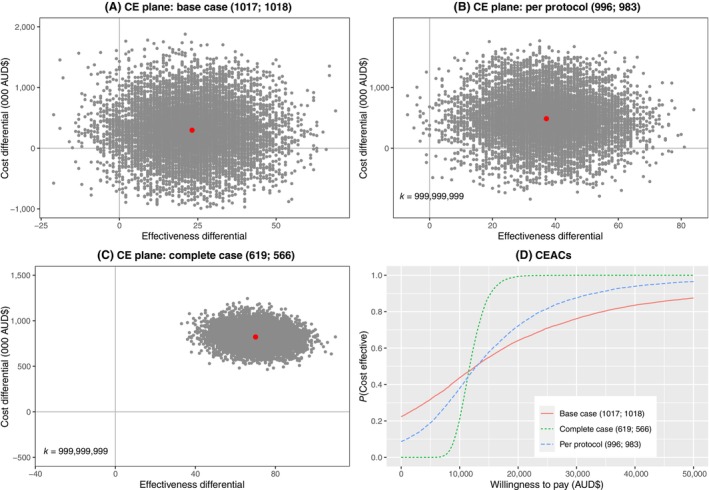
Cost‐effectiveness analyses (cost per surgical site infection (SSI) avoided). Panels (A–C) show cost‐effectiveness (CE) planes for ci‐NPWT (closed incision negative pressure wound therapy) compared to standard dressing for base case, per protocol and complete case analyses respectively. Panel (D) shows corresponding cost‐effectiveness acceptability curves (CEACs). Brackets give sample size: (ci‐NPWT, standard dressing).

Table 1 gives incremental differences in costs and SSI avoided, with uncertainty captured using bootstrapped CIs, for the base case and per protocol and complete case sensitivity analyses. In the base case (ITT), ci‐NPWT reduces SSI number, but with increased costs; however, there is great uncertainty for both costs and effects (wide CIs). The resulting ICER suggests ci‐NPWT costs an additional $12 849 per SSI avoided. Most (75%) bootstrapped cost‐effectiveness estimates fall in the north‐east quadrant of the cost‐effectiveness plane (Fig. 1, panel A), suggesting that ci‐NPWT is likely more effective, but more costly.

**Table 1:** Incremental cost‐effectiveness analysis (CEA) results

Analysis (*n* ci‐NPWT; *n* standard dressing)	Incremental cost (AUD$, 2020)			Incremental SSI avoided			Incremental cost‐effectiveness ratio (ICER)
	Mean	Lower 95% CI limit	Upper 95% CI limit	Mean	Lower 95% CI limit	Upper 95% CI limit	
Base case (1017; 1018)	297 975	−555 590	1 185 943	23.2	−1	48	12 849
Per protocol (996; 983)	486 417	−282 178	1 293 369	37.1	12	62	13 103
Complete case (619; 566)	822 309	630 666	1 019 796	70.1	51	90	11 737

The incremental difference is based on the mean of the bootstrap samples, and the 95% CIs are based on the 2.5th and 97.5th percentiles of the bootstrap samples.

Sensitivity analyses had similar results. The per protocol analysis had a very similar ICER ($13 103 per SSI‐avoided). The complete case analysis has a lower ICER ($11 737 per SSI‐avoided), with all bootstrap estimates falling in the north‐east (more costly, more effective) quadrant.

### Cost‐utility analysis (CUA)

**Figure 2:**
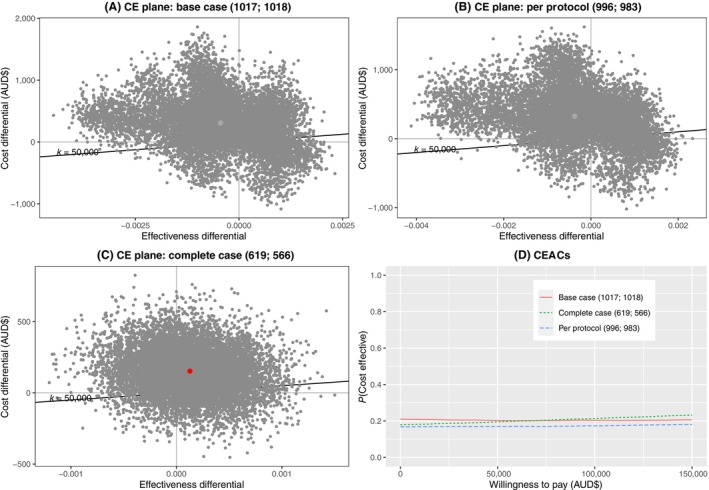
Cost‐effectiveness analyses (cost per quality‐adjusted life years (QALY) gained). Panels (A–C) show cost‐effectiveness (CE) planes for ci‐NPWT (closed incision negative pressure wound therapy) compared to standard dressing for base case, per protocol and complete case analyses respectively. The light grey areas show the cost‐effectiveness acceptability area (eg incremental cost‐effectiveness ratio (ICER) ≤$50 000/QALY). Panel (D) shows corresponding cost‐effectiveness acceptability curves (CEACs). Brackets give sample size: (ci‐NPWT, standard dressing).

Table 2 shows the same quantities as Table 1, but for the CUA. In the base case, standard dressings dominate ci‐NPWT: standard dressing is both less costly and has a greater (though negligible) gain in QALYs. Again, there is great uncertainty around both costs and effects. Most (54%) bootstrapped cost‐effectiveness estimates fall in the north‐west quadrant (Fig. 2 panel A), suggesting that ci‐NPWT is less effective and more costly. At $50 000 per QALY threshold, there is only a 20% chance of ci‐NPWT being cost‐effective (Fig. 2, panel D).

**Table 2:** Incremental cost‐utility analysis (CUA) results

Analysis (*n* ci‐NPWT; *n* standard dressing)	Incremental cost (AUD$, 2020)			Incremental QALYs			Incremental cost‐effectiveness ratio (ICER)
	Mean	Lower 95% CI limit	Upper 95% CI limit	Mean	Lower 95% CI limit	Upper 95% CI limit	
Base case (1017; 1018)	310	−530	1185	−0.0004	−0.0032	0.0015	Dominated
Per protocol (996; 983)	325	−455	1138	−0.0004	−0.0031	0.0014	Dominated
Complete case (619; 566)	153	−171	475	0.0001	−0.0006	0.0009	1 189 243

The incremental difference is based on the mean of the bootstrap samples, and the 95% CIs are based on the 2.5th and 97.5th percentiles of the bootstrap samples.

Sensitivity analyses have broadly similar results. Standard dressings dominate ci‐NPWT (higher QALYs and less costly) in the per protocol analysis. For complete case analysis, ci‐NPWT accrues slightly more QALYs, but the gain is very small (0.0001) and non‐significant (95% CI: −0.0006, 0.0009), and has a large cost of $1 189 243 per‐additional‐QALY.

## Discussion

Our CUA based on rigorous data from DRESSING suggests ci‐NPWT provides no benefit for women in terms of HRQoL and is likely to cost more than standard care, having considered both SSI prevention and treatment costs. Higher costs seem to be driven predominantly by ci‐NPWT intervention costs (Table S1). CUAs are generally preferred over CEAs to inform resource implementation decisions, as QALY outcomes capture HRQoL benefits and the estimated cost‐per‐QALY estimates are comparable between interventions and against standard funding thresholds.

There is a high level of uncertainty in both cost and effect estimates. Nevertheless, taking note of this uncertainty, ci‐NPWT is unlikely to be cost‐effective at conventional willingness‐to‐pay thresholds, with only a 20% chance it is cost‐effective at a conventional threshold of $50 000 per QALY. It also remains unlikely to be cost‐effective at thresholds well in excess of this (up to at least $150 000 per QALY). This finding is robust to assumptions made related to protocol deviation and missing data. While there is increasing clinical evidence around effectiveness of ci‐NPWT for preventing SSI in wounds healing by primary closure following CS, this CUA based on data from the largest randomised controlled trial (RCT) available in this context suggests it should *not* be routinely implemented to prevent SSI in this population.

For completeness, we additionally undertook a CEA, also specified in the trial protocol.^10^ Based on an ITT analysis, we estimate ci‐NPWT to cost an additional $12 849 (95%CI −$62 138 to $133 378) per SSI avoided. Whether this represents acceptable value for money would require knowledge of what society is willing to pay to avoid an SSI, which is unknown. Previous studies have reported SSI to be associated with an additional cost of US$32 187 (approximately AU$42 500) during an index stay, for patients undergoing selected coronary artery bypass, orthopaedic and bariatric surgery procedures.^25^ This might suggest that paying a ‘best estimate’ of $12 849 to avoid an SSI represents acceptable value for money. However, our cost estimates already include the additional costs associated with treating SSIs up to four weeks post‐discharge, as well as prevention costs. Furthermore, other potential costs and benefits (such as discomfort associated with the prevention and treatment of SSI or any impact on ability to care for a newborn infant) have not been considered in the CEA. Thus, we consider the CUA results to be of primary relevance.

Prior to the current study, there was very limited evidence on the cost‐effectiveness of prophylactic ci‐NPWT for wound closure by primary intention. A recent Cochrane review found low to moderate certainty evidence that ci‐NPWT may be cost‐effective in some settings to aid surgical wound healing by primary closure.^7^ Based on two studies the authors found there was moderate level evidence that ci‐NPWT is probably cost‐effective in obese women undergoing CS.^7^ One of these studies was based on a small pilot trial of 87 participants and reported ci‐NPWT to cost AU$42 340 (95% CI dominant to $888 019) per QALY gained.^26^ Hence, while the point estimate suggested ci‐NPWT might be cost‐effective at conventional thresholds, the findings were highly uncertain. The other study was based on a Danish trial of 876 obese women with 30‐day follow‐up post‐CS.^27^, ^28^ The economic study used a three‐month horizon for costs and a one‐year horizon for outcomes (utilising EQ‐5D‐5L to estimate QALYs), and reported ci‐NPWT to be dominant, as it was both less costly and more effective than standard dressing. However, differences in costs and effects were non‐significant. The authors reported ci‐NPWT to have a 92.8% probability of being cost‐effective at a willingness‐to‐pay threshold of Euro 30 000 per QALY. Notably, in the Danish study, the EQ‐5D‐5L values at 30 days were extrapolated to a one‐year time horizon. This effectively assumes a non‐significant QALY difference in favour of ci‐NPWT at 30 days was maintained for a year, a seemingly strong assumption. In addition, a model‐based evaluation undertaken by Tuffaha and colleagues from the perspective of Queensland Health in Australia suggested that NPWT had a 65% likelihood of being cost‐effective compared to standard dressings in preventing SSI in obese women undergoing elective CS. However, they reported considerable uncertainty in the cost‐effectiveness estimate and suggested further research investigate costs and benefits of NPWT in this setting ahead of implementation.^29^ Tuuli and colleagues did not report an economic evaluation alongside their recent RCT, and the negative trial finding (stopped due to futility) did not support the routine use of prophylactic ci‐NPWT in obese women following CS.^8^ Our findings based on an economic evaluation alongside the DRESSING trial are thus important, as they contradict the findings by Hyldig and colleagues,^28^ and Tuffaha and colleagues,^29^ and cast doubt on the value provided by routine prophylactic ci‐NPWT for obese women following CS.

A strength of our study was that it was undertaken alongside a large, prospective RCT that took steps to minimise risks for potential bias. Despite the large cohort, cost‐effectiveness estimates remain uncertain – substantially reducing this is likely to require a prohibitively large trial. It is possible that not all relevant costs and benefits have been captured in the cost‐effectiveness estimate. The evaluation does not capture costs borne by the women themselves such as travel to appointments after discharge, or inconvenience that might be associated with SSIs. We tried to capture any HRQoL benefits but did not observe a difference between groups. While this is consistent with the lack of a difference in HRQoL observed by Hyldig et al. in the Danish study using the EQ‐5D‐5L measure,^27^, ^28^ it is possible that neither the SF‐6D (SF‐12v2) nor EQ‐5D‐5L generic measures are sufficiently sensitive to detect potential differences. Similarly, DRESSING reported a higher incidence of blistering in the ci‐NPWT (4%) than standard dressing (2%) group (RR 1.72, 95%CI 1.04 to 2.85, *P* = 0.034), and additional associated costs may not have been captured. Nevertheless, we consider that costs to the healthcare service associated with blistering would likely be low (beyond any already captured costs – eg antibiotic use). Finally, it is important to note that DRESSING was undertaken within the Australian health system, and associated unit costs utilised; caution is recommended if generalising findings to other settings.

In conclusion, the results of this economic analysis assessed alongside the clinical outcomes from the large randomised controlled DRESSING trial, suggest that using ci‐NPWT to prevent SSI in this population is unlikely to be cost‐effective when compared to standard care, and should not be routinely implemented.

ci‐NPWT, closed incision negative pressure wound therapy; SSI, surgical site infection

ci‐NPWT, closed incision negative pressure wound therapy; QALY, quality‐adjusted life years

## Supporting information


**Appendix S1.** Missing data methodology and analysis; resource data collection and costing detail; univariate cost comparison between arms; utility and QALY by timepoint and arm; CHEERS checklist.
