# Supplementary material for: Cost‐effectiveness of closed incision negative pressure wound therapy in preventing surgical site infection among obese women giving birth by caesarean section: An economic evaluation (DRESSING trial)
Source: Aust N Z J Obstet Gynaecol. 2023 May 18;63(5):673–80. doi: 10.1111/ajo.13677 (PMC10952760; doi:10.1111/ajo.13677)
Supplement: Supplementary file 1 — Appendix S1. Missing data methodology and analysis; resource data collection and costing detail; univariate cost comparison between arms; utility and QALY by timepoint and arm; CHEERS checklist. [file AJO-63-673-s001.docx]

Cost-effectiveness of closed incision negative-pressure wound therapy in preventing surgical site infection among obese women giving birth by caesarean section: an economic evaluation (DRESSING TRIAL)

# Supplementary information

# Method for addressing missing data

Following missing data recommendations for within trial economic evaluations, we explored patterns of missing data (1) and an intention to treat (ITT) analysis rather than a complete case analysis was used for the base-case analysis (2). The mice package (3) within R was used to explore patterns of missingness and conduct multiple imputation (MI). In line with primary analysis from the clinical paper, we singly impute missingness on SSI (the primary outcome; missingness: ci-NPWT=9; standard=19) and assume no SSI for women missing the primary outcome. Subsequently, in the MI that follows, SSI is treated as having no missing data. Conducted separately for each arm (two imputation models, allowing for different patterns of missingness between arms (1)), we created 50 imputed data-sets . Both multiple imputation models included: costs (total for hospital resources, and weekly totals following discharge); outcomes (SSI avoided, baseline and discharge utility); variables significantly (p<0.1) associated with missingness including: BMI group; age; smoking status; presence of respiratory issues; presence of thromboembolytic disease; parity; number of previous CS; American Society of Anaesthesiologists (ASA) status; surgery type [elective/semi-urgent]; surgery time; type of skin closure; use of antibiotics at surgery; wound bleeding recorded in notes; dehisecence recorded in notes; haematoma recorded in notes; seroma recorded in notes); and variables to be included in subsequent regressions (as advised by White, Royston (4); includes recruitment site). Overall, hospital costs and weekly cost totals following discharge were used since the underlying resource items were typically all present or all absent (e.g. when a research nurse could contact participants, all questions were answered). Utility scores (rather than separate items of the SF-12v2) were used for the same reason.

# Analysis of missing data

For the economic analysis, we require data from a larger range of variables than the primary clinical analysis; consequently, incomplete data is common. A simplified graphical representation of missingness is given in Figure S1. Missingness is very low in the data relating to the index admission, but is more common for data collected from the weekly telephone interviews after discharge. Incomplete data for any *one* of these interviews prevents a participant from being in the complete case analysis. As a result of such missing data, the complete case analysis has n=619 and n=566 participants for ci-NPWT and standard dressing arms respectively. The percentage of missing data is significantly different between arms (Fisher’s exact test, p=0.0172): 39% in ci-NPWT versus 44% in standard dressing.

# Table S1: Summary of resource data collection & costing methods

| **Resource measured** | **Unit cost (AUD$, 2020 values)** | **Source for unit cost** |
| --- | --- | --- |
| **I. Interventions** | | |
| NPWT PICO^TM^ unit | 195 | Costs charged to trial |
| Standard dressing | 6.962 | Assumed based on cost of Comfeel Plus^TM^, PBS 2020 ^a^ |
|  |  |  |
| **II. Hospital care** | | |
| Hospital stay for index admission, caesarean section, AR-DRGv10 codes: ^b^ |  | NEPD 2020-21 ^b^ |
| O01A (per stay of 2 to 20 days) | 17,170 |  |
| O01A (per marginal stay day >20 days) | 1,472 |  |
| O01A (per same day discharge) | 4,713 |  |
| O01A (per stay of 1 day) | 10,942 |  |
| O01B (per stay of 1 to 13 days) | 12,312 |  |
| O01B (per marginal day >13 days) | 1,341 (long-stay) |  |
| O01C (per stay of 1 to 9 days) | 10,074 |  |
| O01C (per marginal day >9 days) | 1,522 (long-stay) |  |
|  |  |  |
| Hospital staff consulted in relation to wound during index admission (cost per consultation) | | |
| Doctor | 22.97 | Queensland Government ^c^ |
| Senior doctor (registrar/consultant) | 34.42 | Queensland Government ^d^ |
| Nurse | 12.95 | Queensland Government ^e^ |
| Midwife or Wound clinic nurse | 16.67 | Queensland Government ^f^ |
|  |  |  |
| Medication for treatment of SSI | Varies | PBS 2020 ^g^ |
|  |  |  |
| **III. Post-discharge care (up to 4-week follow up)** | | |
| Consultations with community health staff |  |  |
| General practitioner (GP) visit | 38.20 | Item 23, MBS 2020 ^h^ |
| GP practise nurse, Community nurse | 28.10 | Item 1600, MBS 2020 ^h^ |
| Midwife | 33.30 | Item 82105, MBS 2020 ^h^ |
|  |  |  |
| Hospital use post discharge | | |
| Outpatient attendance | 186.20 | NEPD 2020-21 ^i^ |
| ED visit (non-admitted) | 251.64 | NEPD 2020-21 ^j^ |
| Hospital readmission to AR-DRGv10: ^b^ |  | NEPD 2020-21 ^b^ |
| O061B (per stay of 1 to 7 days) | 3,370 |  |
| O061B (per marginal day above 7 days) | 1,186 (long stay) |  |
|  |  |  |
| Medication for treatment of SSI | Varies | PBS 2020 ^g^ |

^a^ PBS, Pharmaceutical Benefits Schedule, available at <http://www.pbs.gov.au/pbs/home>, accessed 19.11.2020. In particular, based on the “Comfeel Plus Transparent 3533” (<https://www.pbs.gov.au/medicine/item/4907G-4924E-4947J> – considered representative of the standard dressings used in the trial) with DPMQ of $69.62 for a packet of 10. Thus, have assumed an individual unit cost of 69.62/10. All post discharge dressing use are assumed to be standard dressings, since the ci-NPWT PICO device only comes with two NPW bandages.

^b^ The NEPD, National Efficient Price Determination 2020-21 provides inlier weights which apply to all lengths of stay between defined bounds. It was assumed that the full inlier weight applied to hospital stays within this range, and reduced (increased) the cost for shorter (longer) stays by the short-stay (long-stay) outlier per diem. Available at <https://www.ihpa.gov.au/publications/national-efficient-price-determination-2020-21>, accessed 20/5/20.

Australian Refined Diagnosis Groups AR-DRGv10.0 codes are defined as follows:

O01A, Caesarean Delivery, Major complexity

O01B, Caesarean Delivery, Intermediate complexity

O01C, Caesarean Delivery, Minor complexity

O061B, Postpartum and Post Abortion W/O GIs, Minor Complexity

^c^ L5 doctor, from source: annual salary $113,916; hourly rate $70.6667. Assume 15 minute consultation, with 30% oncosts: 70.6667*0.25*1.3. Source: Queensland Government, "Senior medical officers and resident medical officers" – <https://www.health.qld.gov.au/hrpolicies/salary/medical#smormo>, accessed 31.10.2020

^d^ L18 doctor, from source: annual salary $179,703; hourly rate $105.9030. Assume 15 minute consultation, with 30% oncosts: 105.9030*0.25*1.3. Source: Queensland Government, "Senior medical officers and resident medical officers" – <https://www.health.qld.gov.au/hrpolicies/salary/medical#smormo>, accessed 31.10.2020

^e^ Enrolled nurse, nurse grade 3, pay point 3, from source: annual salary $64,208; hourly rate $39.8310. Assume 15-minute consultation, with 30% oncosts: 39.8310*0.25*1.3. Source: Queensland Government "Wage rates - nursing stream" – <https://www.health.qld.gov.au/hrpolicies/salary/nursing>, accessed 31.10.2020

^f^ Registered midwife/nurse, nurse grade 5, pay point 4, from source: annual salary $82,695; hourly rate $51.2991. Assume 15-minute consultation, with 30% oncosts: 51.2991*0.25*1.3. Source: Queensland Government "Wage rates - nursing stream" – <https://www.health.qld.gov.au/hrpolicies/salary/nursing>, accessed 31.10.2020

^g^ PBS, Pharmaceutical Benefits Schedule, available at <http://www.pbs.gov.au/pbs/home>, accessed 01.06.2020

^h^ MBS, Medicare Benefits Schedule version 20April2020, available at <http://www.mbsonline.gov.au/internet/mbsonline/publishing.nsf/Content/Home>, accessed 31.10.2020

^i^ NEPD 2020-21, for non-admitted patients, Tier 2, V5.0, code: 40.13: Wound management

^j^ NEPD 2020-21, for emergency department patients, URG V1.4, code: 118: “N-A_T5_Obstetric illness/Newborn/Neonate”

# Table S2: Costs (2020 AUD$ per person, mean and SD) overall & arm comparison from all available data

|  | **All** | | | **ci-NPWT** | | | **Standard dressing** | | | **Arm comparison** | | | |
| --- | --- | --- | --- | --- | --- | --- | --- | --- | --- | --- | --- | --- | --- |
| **Item** | **N** | **Mean Cost (AUD$)** | **SD** | **N** | **Mean Cost (AUD$)** | **SD** | **N** | **Mean Cost (AUD$)** | **SD** | **Mean differ. (AUD$)** | **95% CI** | | **P-Value** |
| I. Interventions | | | | | | | | | | | | | |
| Standard dressings, hospital | 2,019 | 3.75 | 4.19 | 1,011 | 0.38 | 2.12 | 1,008 | 7.13 | 2.78 | -6.76 | -6.97 | -6.54 | <0.0001 |
| NPWT | 2,019 | 99.38 | 97.51 | 1,011 | 189.79^[[1]](#footnote-2)^ | 31.45 | 1,008 | 8.71 | 40.29 | 181.09 | 177.93 | 184.24 | <0.0001 |
| Standard dressings, PD | 1,264 | 5.15 | 17.42 | 649 | 4.98 | 17.70 | 615 | 5.33 | 17.13 | -0.35 | -2.28 | 1.57 | 0.7176 |
| II. Hospital care | | | | | | | | | | | | | |
| Index admission | 2,019 | 13,300 | 3,618 | 1,011 | 13,317 | 3,948 | 1,008 | 13,282 | 3,256 | 36 | -280 | 352 | 0.8244 |
| Doctor | 2,017 | 0.50 | 10.82 | 1,010 | 0.39 | 4.38 | 1,007 | 0.62 | 14.67 | -0.23 | -1.18 | 0.72 | 0.6347 |
| Senior doctor | 2,017 | 0.49 | 10.07 | 1,010 | 0.51 | 8.99 | 1,007 | 0.48 | 11.06 | 0.03 | -0.85 | 0.91 | 0.9420 |
| Nurse | 2,017 | 0.01 | 0.41 | 1,010 | 0.03 | 0.58 | 1,007 | 0.00 | 0.00 | 0.03 | -0.01 | 0.06 | 0.1574 |
| Midwife | 2,017 | 0.85 | 4.98 | 1,010 | 0.86 | 4.43 | 1,007 | 0.84 | 5.47 | 0.01 | -0.42 | 0.45 | 0.9497 |
| WCN | 2,017 | 0.08 | 1.66 | 1,010 | 0.05 | 1.17 | 1,007 | 0.12 | 2.03 | -0.07 | -0.21 | 0.08 | 0.3694 |
| SSI medication | 2,017 | 6.08 | 38.80 | 1,009 | 6.26 | 38.78 | 1,008 | 5.90 | 38.84 | 0.35 | -3.03 | 3.74 | 0.8374 |
| III. Post-discharge care | | | | | | | | | | | | | |
| GP | 1,256 | 55.10 | 66.05 | 645 | 56.43 | 68.03 | 611 | 53.69 | 63.93 | 2.73 | -4.57 | 10.04 | 0.4629 |
| GP nurse | 1,258 | 0.76 | 7.22 | 647 | 0.69 | 7.13 | 611 | 0.83 | 7.33 | -0.13 | -0.93 | 0.67 | 0.7446 |
| Community nurse | 1,258 | 0.71 | 7.48 | 647 | 0.69 | 7.30 | 611 | 0.74 | 7.68 | -0.04 | -0.87 | 0.79 | 0.9229 |
| Midwife | 1,257 | 14.49 | 37.88 | 647 | 14.51 | 37.27 | 610 | 14.47 | 38.54 | 0.05 | -4.15 | 4.25 | 0.9822 |
| Hospital use post discharge | | | | | | | | | | | | | |
| Out patient attendance | 1,258 | 22.65 | 160.02 | 647 | 16.69 | 95.74 | 611 | 28.95 | 207.32 | -12.26 | -30.30 | 5.78 | 0.1827 |
| ED attendance | 1,258 | 4.00 | 45.82 | 647 | 5.83 | 58.28 | 611 | 2.06 | 26.88 | 3.77 | -1.20 | 8.75 | 0.1370 |
| Readmission | 1,260 | 70.63 | 547.58 | 649 | 74.81 | 553.44 | 611 | 66.18 | 541.70 | 8.63 | -51.91 | 69.17 | 0.7798 |
| SSI Medication | 1,259 | 6.89 | 34.34 | 649 | 5.63 | 29.54 | 610 | 8.24 | 38.78 | -2.61 | -6.44 | 1.22 | 0.1815 |
|  |  |  |  |  |  |  |  |  |  |  |  |  |  |
| Total costs | 1,251 | 13,544 | 2,998 | 644 | 13,622 | 3,127 | 607 | 13,460 | 2,855 | 162 | -170 | 494 | 0.3385 |

# Table S3: Utility & QALY outcomes overall & arm comparison

|  | **All** | | | **ci-NPWT** | | | **Standard dressing** | | | **Arm comparison** | | | |
| --- | --- | --- | --- | --- | --- | --- | --- | --- | --- | --- | --- | --- | --- |
| **SF-12v2** | **N** | **Mean** | **SD** | **N** | **Mean** | **SD** | **N** | **Mean** | **SD** | **Dif.** | **95% CI** | | **P-Value** |
| Baseline utility | 1,940 | 0.74 | 0.13 | 980 | 0.74 | 0.13 | 960 | 0.74 | 0.13 | 0.0000 | -0.0100 | 0.0100 | 0.7017 |
| Post discharge utility, week 1 | 1,771 | 0.75 | 0.14 | 896 | 0.75 | 0.14 | 875 | 0.75 | 0.14 | 0.0000 | -0.0100 | 0.0100 | 0.8278 |
| Post discharge utility, week 2 | 1,671 | 0.81 | 0.13 | 842 | 0.82 | 0.13 | 829 | 0.81 | 0.13 | 0.0000 | -0.0100 | 0.0200 | 0.6457 |
| Post discharge utility, week 3 | 1,630 | 0.84 | 0.12 | 823 | 0.84 | 0.12 | 807 | 0.84 | 0.12 | 0.0000 | -0.0200 | 0.0100 | 0.5209 |
| Post discharge utility, week 4 | 1,650 | 0.87 | 0.11 | 832 | 0.87 | 0.11 | 818 | 0.87 | 0.11 | 0.0000 | -0.0100 | 0.0100 | 0.5910 |
| QALY | 1,198 | 0.07 | 0.01 | 624 | 0.07 | 0.01 | 574 | 0.07 | 0.01 | 0.0000 | 0.0000 | 0.0000 | 0.6549 |

# Table S4: CHEERS checklist. Unless indicated otherwise, page numbers relate to the main text file.

| **Section/item** | **Item No** | **Recommendation** | **Reported on page/section** |
| --- | --- | --- | --- |
| **Title and abstract** | | | |
| Title | 1 | Identify the study as an economic evaluation or use more specific terms such as “cost-effectiveness analysis”, and describe the interventions compared. | p.1 |
| Abstract | 2 | Provide a structured summary of objectives, perspective, setting, methods (including study design and inputs), results (including base case and uncertainty analyses), and conclusions. | p.2 |
| **Introduction** | | | |
| Background and objectives | 3 | Provide an explicit statement of the broader context for the study. | p.3 |
|  |  | Present the study question and its relevance for health policy or practice decisions. | p.3 |
| **Methods** | | | |
| Target population and subgroups | 4 | Describe characteristics of the base case population and subgroups analysed, including why they were chosen. | p.5 |
| Setting and location | 5 | State relevant aspects of the system(s) in which the decision(s) need(s) to be made. | p.4 |
| Study perspective | 6 | Describe the perspective of the study and relate this to the costs being evaluated. | p.4 |
| Comparators | 7 | Describe the interventions or strategies being compared and state why they were chosen. | p.3-4 |
| Time horizon | 8 | State the time horizon(s) over which costs and consequences are being evaluated and say why appropriate. | p.4 |
| Discount rate | 9 | Report the choice of discount rate(s) used for costs and outcomes and say why appropriate. | Noted not applicable, p.4 |
| Choice of health outcomes | 10 | Describe what outcomes were used as the measure(s) of benefit in the evaluation and their relevance for the type of analysis performed. | p.4-5 |
| Measurement of effectiveness | 11a | *Single study-based estimates:* Describe fully the design features of the single effectiveness study and why the single study was a sufficient source of clinical effectiveness data. | p.4 |
|  | 11b | *Synthesis-based estimates:* Describe fully the methods used for identification of included studies and synthesis of clinical effectiveness data. | Not applicable |
| Measurement and valuation of preference based outcomes | 12 | If applicable, describe the population and methods used to elicit preferences for outcomes. | p.4-5 |
| Estimating resources and costs | 13a | *Single study-based economic evaluation:* Describe approaches used to estimate resource use associated with the alternative interventions. Describe primary or secondary research methods for valuing each resource item in terms of its unit cost. Describe any adjustments made to approximate to opportunity costs. | p.4,  Table S1 |
|  | 13b | *Model-based economic evaluation:* Describe approaches and data sources used to estimate resource use associated with model health states. Describe primary or secondary research methods for valuing each resource item in terms of its unit cost. Describe any adjustments made to approximate to opportunity costs. | Not applicable |
| Currency, price date, and conversion | 14 | Report the dates of the estimated resource quantities and unit costs. Describe methods for adjusting estimated unit costs to the year of reported costs if necessary. Describe methods for converting costs into a common currency base and the exchange rate. | p.4 |
| Choice of model | 15 | Describe and give reasons for the specific type of decision-analytical model used. Providing a figure to show model structure is strongly recommended. | Not applicable |
| Assumptions | 16 | Describe all structural or other assumptions underpinning the decision-analytical model. | Not applicable |
| Analytical methods | 17 | Describe all analytical methods supporting the evaluation. This could include methods for dealing with skewed, missing, or censored data; extrapolation methods; methods for pooling data; approaches to validate or make adjustments (such as half cycle corrections) to a model; and methods for handling population heterogeneity and uncertainty. | p.5-6,  Supplementary information |
| **Results** | | | |
| Study parameters | 18 | Report the values, ranges, references, and, if used, probability distributions for all parameters. Report reasons or sources for distributions used to represent uncertainty where appropriate. Providing a table to show the input values is strongly recommended. | Not applicable |
| Incremental costs and outcomes | 19 | For each intervention, report mean values for the main categories of estimated costs and outcomes of interest, as well as mean differences between the comparator groups. If applicable, report incremental cost-effectiveness ratios. | p.7-8,  Tables 1-2, S3 |
| Characterising uncertainty | 20a | *Single study-based economic evaluation:* Describe the effects of sampling uncertainty for the estimated incremental cost and incremental effectiveness parameters, together with the impact of methodological assumptions (such as discount rate, study perspective). | p.14-15, Figures 1-2 |
|  | 20b | *Model-based economic evaluation:* Describe the effects on the results of uncertainty for all input parameters, and uncertainty related to the structure of the model and assumptions. | Not applicable |
| Characterising heterogeneity | 21 | If applicable, report differences in costs, outcomes, or cost-effectiveness that can be explained by variations between subgroups of patients with different baseline characteristics or other observed variability in effects that are not reducible by more information. | Not applicable |
| **Discussion** | | | |
| Study findings, limitations, generalisability, and current knowledge | 22 | Summarise key study findings and describe how they support the conclusions reached. Discuss limitations and the generalisability of the findings and how the findings fit with current knowledge. | p.9-12 |
| **Other** | | | |
| Source of funding | 23 | Describe how the study was funded and the role of the funder in the identification, design, conduct, and reporting of the analysis. Describe other non-monetary sources of support. | Title page document, p.3 |
| Conflicts of interest | 24 | Describe any potential for conflict of interest of study contributors in accordance with journal policy. In the absence of a journal policy, we recommend authors comply with International Committee of Medical Journal Editors recommendations. | Title page document, p.3 |

# **Figure S1**: Simplified representation of missingness in trial data (red represents missing data; blue complete data)

# REFERENCES

1. Faria R, Gomes M, Epstein D, White IR. A guide to handling missing data in cost-effectiveness analysis conducted within randomised controlled trials. Pharmacoeconomics. 2014;32(12):1157-70.

2. Noble SM, Hollingworth W, Tilling K. Missing data in trial-based cost-effectiveness analysis: the current state of play. Health Econ. 2012;21(2):187-200.

3. van Buuren S, Groothuis-Oudshoorn K. mice: Multivariate Imputation by Chained Equations in R. 2011. 2011;45(3):67.

4. White IR, Royston P, Wood AM. Multiple imputation using chained equations: Issues and guidance for practice. Statistics in Medicine. 2011;30(4):377-99.

1. Mean ci-NPWT costs in the ci-NPWT arm are less than the unit cost of $195 as 2.7% (27/1,011; available data) report they did not use any ci-NPWT dressings. Mean ci-NPWT costs in the Standard dressing arm are non-zero as 4.7% (45/1,008; available data) report using an ci-NPWT dressing. [↑](#footnote-ref-2)
